# A Digital Image Correlation (DIC) prototype system for crack propagation monitoring in aircraft assemblies

**DOI:** 10.12688/openreseurope.14599.2

**Published:** 2022-09-28

**Authors:** LiKang Luan, Liam Crosbie, Silvain Michel, Erwin Hack

**Affiliations:** 1 Dantec Dynamics GmbH, Ulm, 89077, Germany; 2 Empa, Swiss Federal Laboratories for Materials Testing and Research, Dübendorf, 8600, Switzerland

**Keywords:** DIC, Structural Health Monitoring, SHM, aircraft, aerospace, fatigue, strain

## Abstract

**Background:** In the Clean Sky 2 project DIMES, the cyclic loading of a section of an A320 wing with pre-existing damage was carried out.

**Methods: **We present a Digital Image Correlation (DIC) prototype system to monitor crack propagation in the aircraft wing. This system includes a mount for easy installation and adjustment in a confined space.

**Results: **Strain localization and evaluation due to crack propagation was successfully observed in the Region-of-Interest (ROI) during cyclic fatigue loading. The results from the DIC prototype system were supported by conventional contact Resistance Strain Gauge (RSG) sensors acting as a far-field monitor.

**Conclusions: **Future improvements, the combination of two DIC modules for a stereo DIC system and the potential of the DIC system for ground-based tests and Structural Health Monitoring (SHM) applications are also discussed.

## Introduction

Structural Health Monitoring (SHM) refers to continuous monitoring of engineering structures with various sensor systems and the development of a damage detection strategy ^1^. SHM systems can ensure increased safety and reliability of aircrafts while reducing maintenance costs ^2^. There is an increasing body of research and study of sensor systems with the potential for SHM applications ^3– 7^, including fibre optical systems ^8, 9^. However, in-flight demonstrations of SHM systems are still rare and mostly limited to fibre optical systems ^10^. The DIMES project – Development of Integrated Measurement Systems – aimed at developing an advanced integrated measurement system that has the capability to detect a crack or delamination in a metallic or composite aircraft structure. It is a modular system that accepts diverse sensors such as fibre optics, strain gauges, visual and infrared cameras ^11^. Digital Image Correlation (DIC) ^12, 13^ is a camera-based method that analyzes the deformation of a specimen surface by comparing a series of images before and after deformation, which is now widely used for full-field displacement and strain measurement. Compared to conventional contact sensor systems, the DIC method has a number of advantages such as: non-contact optical measurement, convenient installation and full-field deformation analysis, all of which provide great potential for its use in SHM applications. Hoult *et al.* ^14^ used DIC to measure and characterize cracks in concrete structures. DIC offered a significant advantage over traditional instruments because *a priori* knowledge of the crack locations was not required in the analysis. Sabato *et al.* ^15^ studied the performance of a 3D-DIC system for railroad tie inspection and ballast support assessment. They found that the high-rate and non-contacting nature of the optical measurement approach led to more frequent and cost-effective measurements, compared to most of the conventional SHM sensing systems such as strain gauges and accelerometers, which are limited due to wires, data transmission, power requirement. Ngeljaratan *et al.* ^16^ researched the potential of applying DIC systems to the structural vibration behaviour of bridges. They found that very comparable results are obtained from DIC and conventional instrumentation, which showed the potential of DIC to be integrated into larger SHM and condition assessment frameworks.

In this work, the DIC method was used to monitor crack initiation and propagation in an aircraft wing during cyclic fatigue and flight-cycle loading simulating in-flight conditions. A prototype DIC system specially designed for this application is described, which was installed in a section of a wing, and used to monitor several Regions of Interest (RoI). The test results from the prototype DIC system were analyzed and compared with results collected by a remote resistance strain gauge (RSG) sensor for verification of the DIC system. The advantages of the DIC prototype system and its application potential in an in-flight measurement are discussed.

## Methods

### 1. Test setup

**Figure 1.**
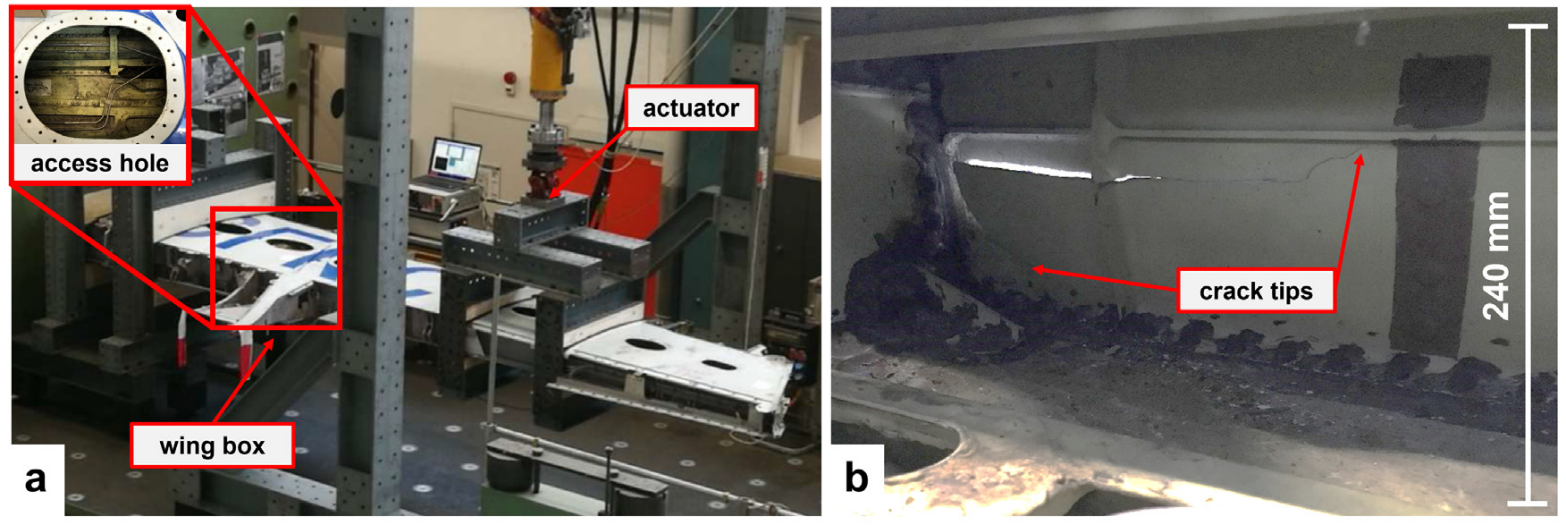
Test setup for the wing section: ( **a**) load frame installed on a strong floor at Empa with an access hole used for installation of the DIC system into the wing box; ( **b**) front spar surface with pre-existing cracks viewed from inside of the wing box.

In the DIMES project, the cyclic loading of a damaged section of an A320 wing was carried out in the laboratories of Empa. The loading concept was inspired by the typical wing loading in service, e.g. bending in both upward and downward directions. To achieve this loading regime, an asymmetric three-point bending setup was implemented as shown in Figure 1(a). The section of wing was placed upside down approximately 1.25 m above a strong floor. Two pairs of wooden blocks fixed the section of wing at one end. The bending moment was applied via a servo-hydraulic actuator mounted towards the wing tip. The loading force was distributed to two ribs via a fixed frame. The front spar of the wing, shown in Figure 1 (b), contained a pre-existing crack which was defined as the RoI. The DIC system was installed into the wing box through an access hole as highlighted in Figure 1(a). The inside surface of the front spar was monitored for the detection of any crack propagation during the cyclic loading of the section of wing. Conventional contact sensors such as resistance strain gauges (RSG) and Fiber Bragg Gratings (FBG) were installed on the wing section. The strain value of one RSG installed on the outside surface of the front spar towards the clamp was used for comparison with the DIC test results.

### 2. Design and installation of prototype DIC system

**Figure 2.**
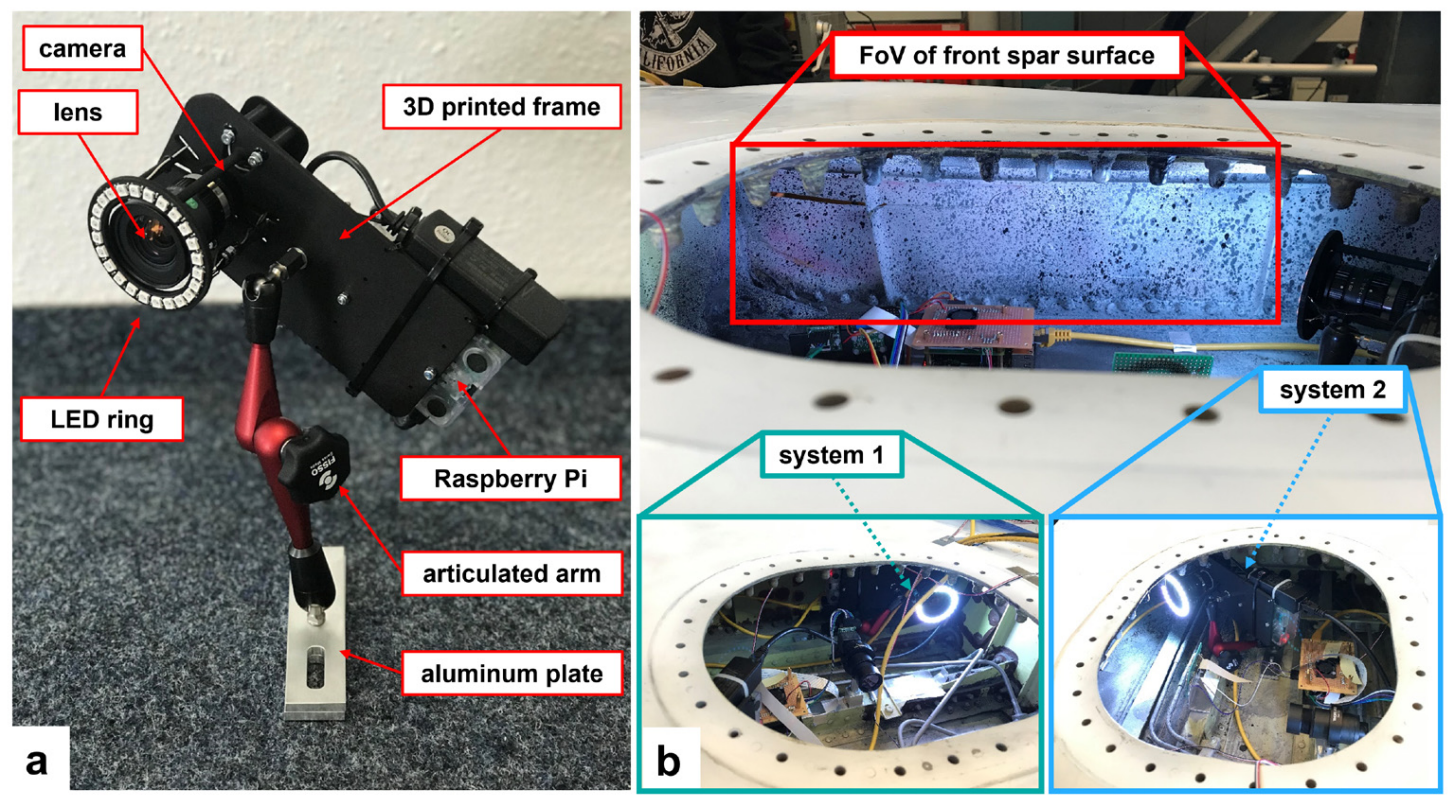
( **a**) the prototype DIC system with its ring LED. ( **b**) the installation of DIC system 1 perpendicular to the front spar surface and DIC system 2 with an oblique viewing angle. Other components inside the wing box belong to different measurement systems, which are not discussed here.

A prototype system was designed for this application as shown in Figure 2 (a). The prototype consisted of a VC MIPI IMX296 camera (1440 × 1080 pixels, pixel size 3.45 µm, Vision Components, Ettlingen, Germany), a 5 mm C-Mount Computar lens H0514MP2 and a Raspberry Pi 3B+ (Raspberry Pi Foundation, Cambridge, UK) mounted on a 3D-printed frame with a footprint of 90 mm × 170 mm. The 3D-printed frame was connected to an articulated arm of length 200 mm S-20 FISSO (Baitella AG, Zurich, Switzerland) that had three joints to adjust the viewing angle and working distance of the DIC system relative to the surface of the RoI. The other end of the articulated arm was fixed to a slotted aluminum plate, which in turn was fixed via two Studs from a click bond (Click bond Inc, Carson City, USA) on flat surfaces inside the wing bay. An LED ring (Adafruit Industries, New York, USA) was installed around the lens for illumination in the confined space. The weight of the DIC module, without the cable connections, was less than 750 g. The DIC system was connected to a Network Attached Storage (NAS), where all recorded images were stored together with data from other sensor systems. Post-processing was used for DIC data evaluation, which means that the full-field DIC results were analyzed after the cyclic loading test and subsequently compared with data from a RSG installed on the front spar to verify the performance of the prototype DIC system.

**Figure 3.**
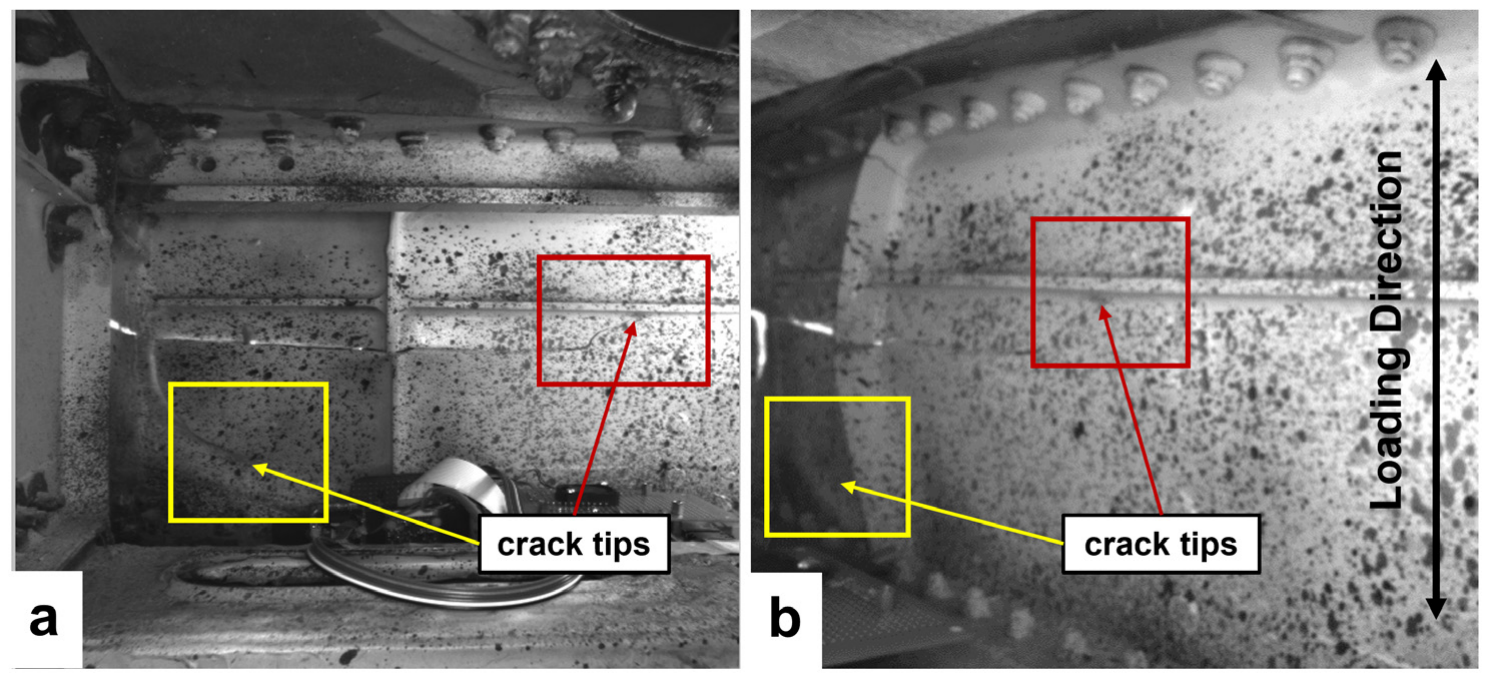
FoV of DIC system 1 ( **a**) and DIC system 2 ( **b**) containing two crack tips.

Spray paint was used to prepare a speckle pattern on the front spar due to its flexible application in the confined space of the wing box. White matte color was first painted as background and black matte color was then sprayed to generate speckle patterns on the front spar surface. Two of the prototype DIC systems were installed in the wing to monitor the same surface area of the front spar as shown in Figure 2 (b). DIC system 1 was aligned perpendicular to the front spar surface and system 2 was installed with an oblique viewing angle. Based on a working distance of approximately 400 mm, a Field-of-View (FoV) of 400 mm × 300 mm was achieved, which allowed the whole of the pre-existing cracks on the front spar to be viewed (see highlighted boxes in Figure 3). One crack tip was located in the left corner of the front spar and the other crack was close to a stringer in the right part of the FoV. After the installation of both DIC systems, the access hole was covered and the LED rings provided sufficient lighting for the DIC measurements. 

### 3. Test method

A cyclic loading test was carried out on the section of wing with a deflection of -80 mm to the fixed wing end and an amplitude of 15 mm at 1.25 Hz via the servo-hydraulic actuator. The image acquisition rate of both DIC systems was set to 0.067 Hz (one image every 15 seconds). The cyclic loading test lasted for around 25 hours and both DIC systems acquired more than 6000 images during the test. The loading direction is indicated in Figure 3. After the cyclic loading test, post-processing of the recorded images from both DIC systems was performed with a facet size of 25×25 pixels and a grid spacing of 10 pixels using Istra4D, Dantec Dynamics’ DIC software. The distribution and magnitude of the maximum principal strain, *ε*_1_, which represents the largest tensile deformation on the front spar surface, was analyzed with a focus on the crack tips, to study the crack propagation during the cyclic loading test.

## Results and discussion

**Figure 4.**
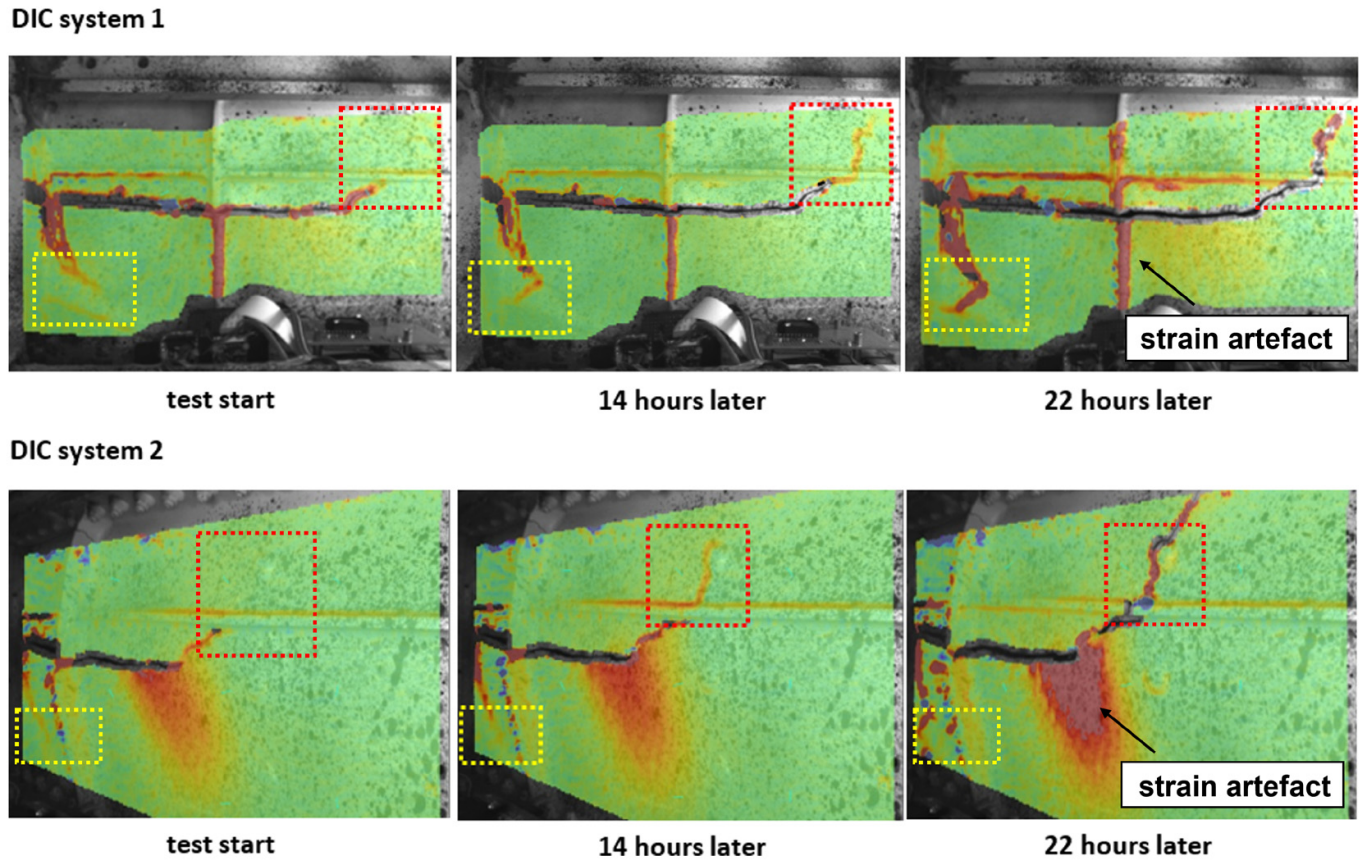
The distribution of the maximum principal strain, *ε*_1_ from both DIC systems at various time steps. Crack propagation was detected in the red box, while crack initiation occurred in the yellow box. It grew perpendicular to the orientation of the existing crack. Note that strain artefacts are highlighted with arrows.

In this study, we used the development of strain localization as a qualitative mapping to evaluate the crack propagation process. Hence, no quantitative strain evaluation and analysis was included. Figure 4 shows the distribution of the (pseudo) maximum principal strain, *ε*_1_ from both DIC systems at various time steps ^17^.

Strain localization was observed around both crack tips in the highlighted boxes shown in Figure 4 at the beginning of the cyclic loading test. After 14 hours, growth of the strain localization was simultaneously observed in both highlighted boxes. After 22 hours, crack propagation of both crack tips on the front spar of the wing was confirmed. Decorrelation of DIC data around the crack tip in the red frame highlighted in Figure 4 was observed due to the crack opening. Other areas of strain localization, highlighted in Figure 4 with arrows, were considered to be strain artefacts, such as at the edge of the correlation area or along lines of structural reinforcement. A large area of strain localization was observed in the middle of the FoV of DIC system 2 in Figure 4, where strain increased during the test. Meanwhile, no strain localization in the same area was detected by DIC system 1, which suggested the strain localization observed in DIC system 2 was also an artefact. It was caused by the out-of-plane motion of the front spar during the cyclic loading test. Sutton *et al.* ^18^ found that out-of-plane motion can introduce displacement gradients in the image plane and results in strain artefacts. Using two DIC systems with different viewing angles to monitor the same region of interest made it possible to identify the influence of out-of-plane motion on the 2D-DIC results. However, the absolute strain value cannot be accurately determined due to the inevitable out-of-plane motion. Therefore, for damage monitoring in this study, we focussed on relative changes in the strain field instead of quantitative strain analysis. Stereoscopic DIC measurement was not considered, as the fields of view and focus changed during the fatigue cycle, and the two DIC systems were mounted independently from each other. However, combining two 2D-DIC systems into a stereo system on a rigid mount is straightforward.

**Figure 5.**
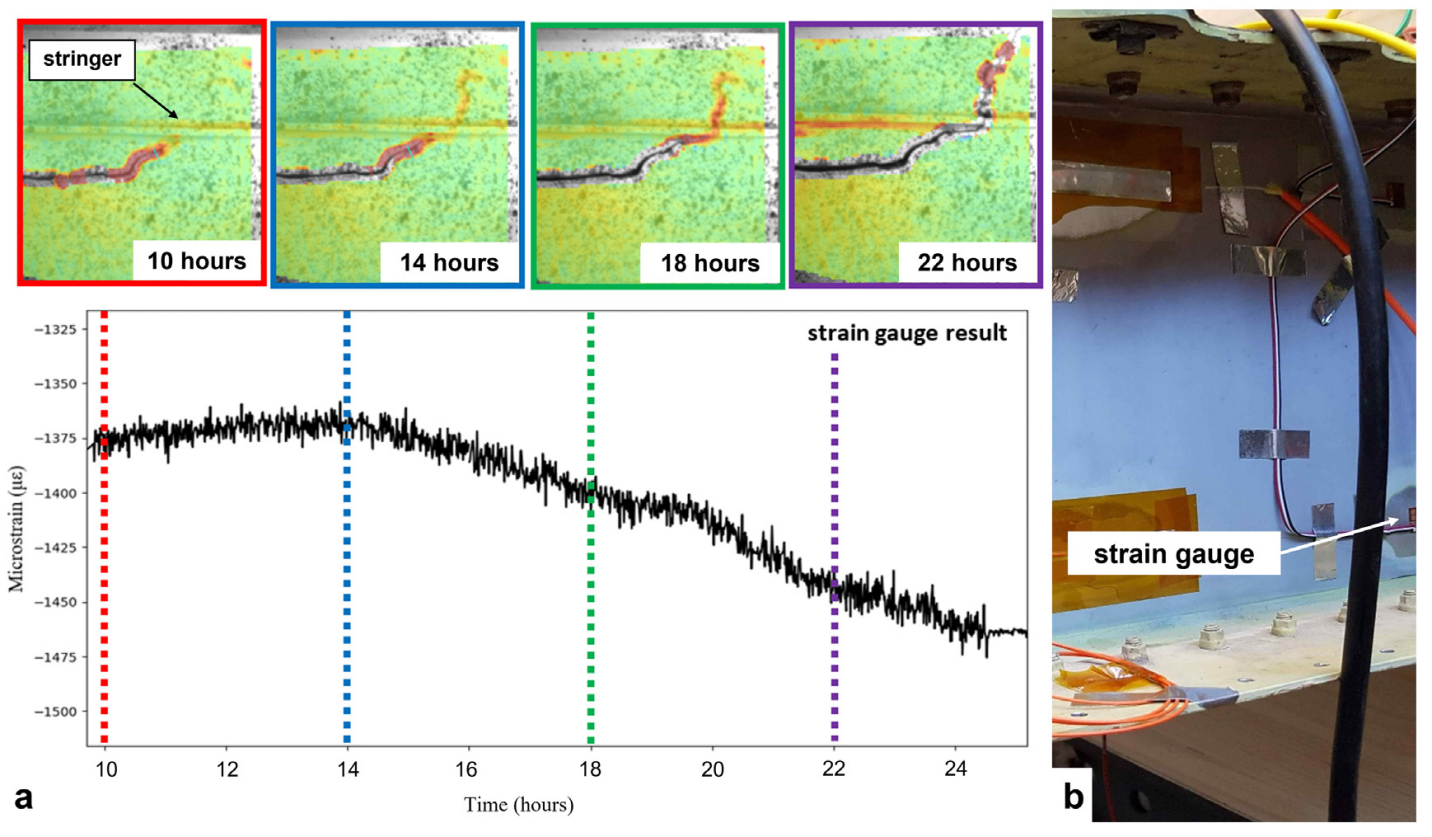
( **a**) Strain signal measured from a RSG sensor installed on the front spar collated with DIC results at various points in time. ( **b**) Position of the strain gauge close to the wooden block used for clamping the wing box.

The RSG installed on the front spar towards the clamp, Figure 5(b) served as a far-field strain monitor of damage propagation. Decreasing levels of compressive strain indicate a strain relieve around the strain gauge, caused by the propagating damage, but it cannot tell the location where this occurs. The DIC results for the strain distribution around the crack tip locate the propagating damage, and therefore they were compared with the results from the RSG at four time steps in Figure 5(a).

The upper part of Figure 5(a) shows the strain evaluation at the crack tip at different time steps in the red highlighted box from DIC system 1 in Figure 4. The bottom part of Figure 5(a) shows the strain value over time measured from the RSG. The RSG signal was captured with a frequency of 7 Hz and was averaged in this graph by 256-value rolling mean to reduce the influence of the cyclic wing deflection on the strain value caused by the fatigue load. During the first hours after the start of the various tests, there was no significant change in the RSG signal or the DIC strain distribution. The crack tip was observed close to the stringer as highlighted in Figure 5(a) with an arrow. A similar strain localization around the crack tip can be observed in strain map at the start of the experiment shown in Figure 4 (DIC system 1). Between 14 and 18 hours, the area of high strain moved from the crack tip across the stringer suggesting crack propagation had begun. Meanwhile, the RSG showed increasing compressive strain. This process continued, and from 22 hours onward, the crack across the stringer was visible and propagated out of the FoV to the top right, while the compressive strain value of the RSG kept increasing. Based on the correlation between data from DIC and RSG, we found that the prototype DIC system exhibited an excellent performance for damage detection and stability in tests over long times. In addition to the onset of damage propagation, the full-field deformation maps from DIC provided information about the location of the damage in a defined RoI. The adaptable mounting would allow this FoV to be changed for either following the crack tip further, or for pointing at other locations where crack initiation was expected.

Nevertheless, there are multiple opportunities for optimization of the prototype DIC system for potential SHM applications. One major downside of the DIC measurements is the relative large volume of data generated, i.e., the images acquired from the cameras. Here, we used 1.5 MB for one image, which added up to 17 GB of data over 24 hours with two systems. One option for data reduction is to use signals from other sensor systems to trigger fast image acquisition by the DIC system for more detailed damage monitoring only when an indication of damage growth is found, while a low image acquisition rate is used otherwise. Based on this method, it would be possible to achieve reduced data volumes and yet still obtain the possibility for detailed analysis of damage evaluation.

Post-processing of recorded images was used in this study, which means there was no real-time feedback for automatic damage detection in the wing from the DIC system. For long-term continuous SHM applications, a real-time measurement capability for the DIC system would be particularly important in order to gain the latest status of the structure. However, automatic damage detection is challenging due to the complexity of damage definition, i.e, the type of damage, the location of damage, and the critical level of damage.

The DIC prototype system tested in this study is a 2D-DIC system, which means strain artefacts caused by out-of-plane deformation are unavoidable. This will raise the risk that accurate information cannot be identified from the artefacts. The development of a 3D-DIC system for this application could solve this problem. The shape and displacement of the specimen surface could be determined so that artefacts could be eliminated ^19^. But there are multiple challenges, such as system calibration in a confined space, the requirement for system stability to maintain a fixed relative position between the two cameras. It would also add weight to the system, which is unwanted in many SHM applications.

## Conclusions

In this work, we have designed a prototype DIC system for crack propagation monitoring in an aircraft wing. Two DIC systems were installed in a wing box and both DIC systems successfully provided data fields that illustrated crack propagation using post-processing after a cyclic loading test. Test results from the DIC systems and an RSG showed consistency with each other during crack propagation. Influence of strain artefacts caused by out-of-plane motion and the optimization of the prototype DIC system have been discussed. A full-field DIC prototype system could provide more detailed damage analysis, such as determination of the location and type of damage, and has potential in SHM applications. 

## Data Availability

Zenodo: DIC images for DIMES paper, https://doi.org/10.5281/zenodo.7082028 This project contains the following underlying data ^17^: -   DIC_System_1_0_hours.bmp -   DIC_System_1_14_hours_later.bmp -   DIC_System_1_22_hours_later.bmp -   DIC_System_2_0_hours.bmp -   DIC_System_2_14_hours_later.bmp -   DIC_System_2_22_hours_later.bmp -   Video_DIC_System_1_EngPrinpicalStrain1.avi -   Video_DIC_System_2_EngPrincipalStrain2.avi Data are available under the terms of the Creative Commons Attribution 4.0 International license (CC-BY 4.0).
