# Supplementary material for: A Digital Image Correlation (DIC) prototype system for crack propagation monitoring in aircraft assemblies
Source: Open Res Eur. 2022 Sep 28;2:82. Originally published 2022 Jun 22. [Version 2] doi: 10.12688/openreseurope.14599.2 (PMC10445805; doi:10.12688/openreseurope.14599.2)
Supplement: Peer review history

# Peer review: Reviewer response for version 2

**DOI:** 10.21956/openreseurope.16352.r30167

**Published:** 2022-10-03

**Authors:** Umberto Galietti

**Affiliations:** 1 Department of Mechanics Mathematics and Management (DMMM), Politecnico di Bari, Bari, Italy

The most important suggestions were well considered and the amended version is fully approved. Excellent piece of work.

 Hopefully there will be chance for the authors in future works to perform a sensitivity analysis and a comparison with other techniques.

Is the study design appropriate and does the work have academic merit?

Yes

Is the work clearly and accurately presented and does it cite the current literature?

Yes

If applicable, is the statistical analysis and its interpretation appropriate?

Not applicable

Are all the source data underlying the results available to ensure full reproducibility?

Partly

Are the conclusions drawn adequately supported by the results?

Yes

Are sufficient details of methods and analysis provided to allow replication by others?

Partly

Reviewer Expertise:

Experimental Stress Analysis; Materials characterization including Fracture Mechanics and Fatigue ; Non Destructive Testing; Infrared Thermography applications.

I confirm that I have read this submission and believe that I have an appropriate level of expertise to confirm that it is of an acceptable scientific standard.

# Peer review: Reviewer response for version 1

**DOI:** 10.21956/openreseurope.15765.r29584

**Published:** 2022-08-18

**Authors:** Umberto Galietti

**Affiliations:** 1 Department of Mechanics Mathematics and Management (DMMM), Politecnico di Bari, Bari, Italy

This is a very nice application of DIC for SHM.

 For the aim of the work, that is showing the feasibility of DIC on a confined volume during a fatigue test, literature provided is a sufficient state of the art. I would suggest anyway to insert some info and citation of the competing SHM systems to enhance to analysis of the system for SHM.

 The declared aim of the prototype is partly demonstrated, being the cracks identified already well grown at the beginning of the test. The capability to identify the start of the crack has still to be experimentally showed. Even if I agree that the system shows enough sensitivity to do it, the demonstration of the capability to identify crack just initiated would increase significantly the advantage of the system for SHM. In fact the SG results already provide info about the occurring damage after 14 hours testing and, if the advantage of the DIC is to provide the info of the damage at an early stage, at least an estimation of the smallest crack for each setup that can be identified should be provided and it can greatly increase the appeal for the use of the prototype.

 In order to enhance the replicability of the test, even if a commercial software was used for this work, I strongly suggest to provide info about the DIC analysis parameters.

 I could not open the *.avi files provided in the repository. For sure it is just because of the programmes that I do not have on the PC, but I suggest anyway to indicate a suitable program to open them.

 For future work a comparison with other cited  techniques and a sensitivity analysis would greatly enhance vantages and disadvantage for SHM competing techniques.

 Thanks for the very nice application.

Is the study design appropriate and does the work have academic merit?

Yes

Is the work clearly and accurately presented and does it cite the current literature?

Yes

If applicable, is the statistical analysis and its interpretation appropriate?

Not applicable

Are all the source data underlying the results available to ensure full reproducibility?

Partly

Are the conclusions drawn adequately supported by the results?

Yes

Are sufficient details of methods and analysis provided to allow replication by others?

Partly

Reviewer Expertise:

Experimental Stress Analysis; Materials characterization including Fracture Mechanics and Fatigue ; Non Destructive Testing; Infrared Thermography applications.

I confirm that I have read this submission and believe that I have an appropriate level of expertise to confirm that it is of an acceptable scientific standard, however I have significant reservations, as outlined above.

# Response

**Published:** 2022-09-14

**Authors:** Erwin Hack

**Affiliations:** Swiss Federal Laboratories for Materials Testing and Research, Switzerland

This is a very nice application of DIC for SHM.

**Thank you for your positive rating**

 For the aim of the work, that is showing the feasibility of DIC on a confined volume during a fatigue test, literature provided is a sufficient state of the art. I would suggest anyway to insert some info and citation of the competing SHM systems to enhance to analysis of the system for SHM.

**We have added some information to the cited literature [14-16] in the Introduction.**  

 The declared aim of the prototype is partly demonstrated, being the cracks identified already well grown at the beginning of the test. The capability to identify the start of the crack has still to be experimentally showed. Even if I agree that the system shows enough sensitivity to do it, the demonstration of the capability to identify crack just initiated would increase significantly the advantage of the system for SHM.

**We have amended the text to clarify that in Fig. 4 we see both crack propagation (red box), as well as crack initiation (yellow box).**

 In fact the SG results already provide info about the occurring damage after 14 hours testing and, if the advantage of the DIC is to provide the info of the damage at an early stage, at least an estimation of the smallest crack for each setup that can be identified should be provided and it can greatly increase the appeal for the use of the prototype.

**Thank you for your suggestion. We agree that, for a practitioner, the minimum detectable crack length is a desirable measure. However, it was not possible to relate the strain concentration to a crack length.**

 In order to enhance the replicability of the test, even if a commercial software was used for this work, I strongly suggest to provide info about the DIC analysis parameters.

**We have added "with a facet size of 25×25 pixels and a grid spacing of 10 pixels" at the appropriate location.**

 I could not open the *.avi files provided in the repository. For sure it is just because of the programmes that I do not have on the PC, but I suggest anyway to indicate a suitable program to open them.

**We are sorry that you could not play the videos. We now provide both videos also in .mp4 format through the repository.**

 For future work a comparison with other cited  techniques and a sensitivity analysis would greatly enhance vantages and disadvantage for SHM competing techniques. Thanks for the very nice application.

**Thank you for this suggestion. However, we feel that it is beyond the scope of the present paper, as we wanted to demonstrate the potential of the DIC prototype.**

# Peer review: Reviewer response for version 1

**DOI:** 10.21956/openreseurope.15765.r29582

**Published:** 2022-08-08

**Authors:** Rachel A. Tomlinson

**Affiliations:** 1 Department of Mechanical Engineering, University of Sheffield, Sheffield, UK

This paper is a well-written account of small, lightweight DIC system which is shown to be effective in an enclosed area such as inside an aircraft wing box for structural health monitoring. The authors describe the new system and present the results of a test which demonstrates its capabilities and limitations.

 I recommend a number of changes to the paper which will clarify what was done to the reader and correct minor errors.

**Introduction**:

 In the introduction the authors state that “in-flight demonstrations of SHM systems are still rare and mostly limited to fibre optical systems” but don’t specifically say that the work presented here (part of the DIMES project) is novel because it can be used in-flight. I think adding this emphasis would highlight the novelty of the work.

**Figures**

 Including photographs of experimental apparatus in papers is always tricky because the authors know exactly what the picture represents, but sometimes forget that the reader does not. All the figures would benefit from some sort of scale indicator and labels on the salient features.

 Figure 1: a) label the wing box, the actuator, the access hole, b) pre-existing cracks. Figure 2: a) label all the features mentioned in the text b) it is really difficult to understand what I’m looking at here- there appears to be other cameras in the image in addition to the DIC?

 It is not clear exactly where the strain gauge is located that is used in Figure 5. Please add a label on one of the images of the apparatus. It is also not clear how the data from this strain gauge compares to the DIC results. Clarification of what you are comparing and why is needed.

 Figure 3: it’s not easy to see the dotted boxes or cracks (I printed the paper in black and white to read it- then had to resort to downloading and enlarging the figures because I couldn’t see the cracks). Suggest you make the boxes white not red and point the arrow actually at the crack tip and not just the box.

 Figures 4 and 5 are maps of principal strain but have no quantitative colour scale. I understand why this is the case, but the reasons should be clearer. I think you need to clarify at the very start of the Results and Discussion section that you are using the strain as a damage mapping technique and not as a quantitative strain technique. Then go on to say you know you have out of plane strain which is causing the spurious strain artefacts, but it will also cause errors in the quantitative 2D strains. You quite rightly say that 3D DIC will be complicated to implement and calibrate in a small space and I honestly think that using 2D DIC works well as a damage tracking method. In addition to a clarification at the start of the Results section, I suggest that after the sentence “Therefore, for damage monitoring, we focussed on relative changes in the strain field in this study” you could add that for this reason there are no quantitative scales in the data images.

 The narrative around Figure 5 is a little confusing. You state that “During the first 10 hours after the start of the experiment, there was no significant change in the RSG signal or the DIC strain distribution” but you don’t actually present these data. I suggest including the data from just after the start of the test to support this statement.

 Replace “After 14 hours” with “Between 14 and 18 hours”.

 You say “Based on the comparison between data from DIC and RSG, we found that the prototype DIC system exhibited an excellent performance and stability in tests over long time periods for damage detection.” I can accept the stability claim, but what in the comparison indicates “excellent performance”? This is too vague, and you could be ore specific in your claims. It would help if you made it clear (as stated above) exactly what you are comparing and why. Are you basically saying that the DIC data shows the cracks growing and the magnitude strain increasing, and the RSG shows strain increasing too? The cynic would then say that you don’t need the complication of DIC. So I think you can emphasise more strongly that, while the RSG gives the same indication of increasing damage through the strain magnitude increase, that ( superior to RSG) the DIC allows location detection also.  Just the addition of a couple of words here would emphasise the advantages of DIC for this sort of work. Also please be consistent with terminology – suggest you refer only to “increasing compressive strain” and don’t then talk about decreasing strain.

 Final line of Results and discussion section: never start a sentence with “And”.

 I think the above recommendations would give the paper polish and make it easier to understand.

Is the study design appropriate and does the work have academic merit?

Yes

Is the work clearly and accurately presented and does it cite the current literature?

Yes

If applicable, is the statistical analysis and its interpretation appropriate?

Not applicable

Are all the source data underlying the results available to ensure full reproducibility?

Yes

Are the conclusions drawn adequately supported by the results?

Partly

Are sufficient details of methods and analysis provided to allow replication by others?

Partly

Reviewer Expertise:

Experimental Mechanics, DIC, strain measurement

I confirm that I have read this submission and believe that I have an appropriate level of expertise to confirm that it is of an acceptable scientific standard.

# Response

**Published:** 2022-09-14

**Authors:** Erwin Hack

**Affiliations:** Swiss Federal Laboratories for Materials Testing and Research, Switzerland

This paper is a well-written account of small, lightweight DIC system which is shown to be effective in an enclosed area such as inside an aircraft wing box for structural health monitoring.

**Thank you for your overall positive rating.**

*Introduction:*

 In the introduction the authors state that “in-flight demonstrations of SHM systems are still rare and mostly limited to fibre optical systems” but don’t specifically say that the work presented here (part of the DIMES project) is novel because it can be used in-flight. I think adding this emphasis would highlight the novelty of the work.

**We have added a phrase in the Introduction to highlight that the work is for simulating in-flight conditions, and that it has advantages compared to FBG.**

*Figures*

 Including photographs of experimental apparatus in papers is always tricky because the authors know exactly what the picture represents, but sometimes forget that the reader does not. All the figures would benefit from some sort of scale indicator and labels on the salient features.

 Figure 1: a) label the wing box, the actuator, the access hole, b) pre-existing cracks. We have added labels to the main parts of the test set-up in Fig.1, as well as a scale bar inside the wing box.

**The figure caption has been updated.**

   Figure 2: a) label all the features mentioned in the text b) it is really difficult to understand what I’m looking at here- there appears to be other cameras in the image in addition to the DIC?

**We have added labels to the main parts of the test set-up in Fig.2 and have highlighted the DIC systems. The caption has been updated.**

 It is not clear exactly where the strain gauge is located that is used in Figure 5. Please add a label on one of the images of the apparatus. It is also not clear how the data from this strain gauge compares to the DIC results. Clarification of what you are comparing and why is needed.

**The RSG in question is located on the front spar towards the clamp of the wing. This location is not visible on any of the figures so far, but we added a photo to Fig. 5. We have added a sentence to clarify the idea of the comparison of the strain values with the DIC images. The caption of Fig 5 was adapted accordingly.**

 Figure 3: it’s not easy to see the dotted boxes or cracks (I printed the paper in black and white to read it- then had to resort to downloading and enlarging the figures because I couldn’t see the cracks). Suggest you make the boxes white not red and point the arrow actually at the crack tip and not just the box

**We have optimized Fig. 3 with full lines and different colours, so that the boxes and arrows are better visible in both colour and gray print. The arrows point directly on the crack tips.**

 Figures 4 and 5 are maps of principal strain but have no quantitative colour scale. I understand why this is the case, but the reasons should be clearer. I think you need to clarify at the very start of the Results and Discussion section that you are using the strain as a damage mapping technique and not as a quantitative strain technique. Then go on to say you know you have out of plane strain which is causing the spurious strain artefacts, but it will also cause errors in the quantitative 2D strains. You quite rightly say that 3D DIC will be complicated to implement and calibrate in a small space and I honestly think that using 2D DIC works well as a damage tracking method. In addition to a clarification at the start of the Results section, I suggest that after the sentence “Therefore, for damage monitoring, we focussed on relative changes in the strain field in this study” you could add that for this reason there are no quantitative scales in the data images.

**Thank you for your suggestion. The following sentences at the beginning of Results and Discussion and further down were added: “In this study, we use the development of strain localization as a qualitative mapping to evaluate the crack propagation process. Hence, no quantitative strain evaluation and analysis is included.” “Therefore, for damage monitoring in this study, we focused on relative changes in the strain field instead of quantitative strain analysis.”**

 The narrative around Figure 5 is a little confusing. You state that “During the first 10 hours after the start of the experiment, there was no significant change in the RSG signal or the DIC strain distribution” but you don’t actually present these data. I suggest including the data from just after the start of the test to support this statement.

**We have shown the strain distribution at test start in Figure 4 – DIC system 1, which shows a similar strain distribution compared to the test result after 10 hours. We added the following sentence:  “A similar strain localization around the crack tip can be observed in strain map at the start of the experiment shown in Figure 4 (DIC system 1).**

 "Replace “After 14 hours” with “Between 14 and 18 hours”. **We have implemented this change to the text.**

 You say “Based on the comparison between data from DIC and RSG, we found that the prototype DIC system exhibited an excellent performance and stability in tests over long time periods for damage detection.” I can accept the stability claim, but what in the comparison indicates “excellent performance”? This is too vague, and you could be ore specific in your claims. It would help if you made it clear (as stated above) exactly what you are comparing and why. Are you basically saying that the DIC data shows the cracks growing and the magnitude strain increasing, and the RSG shows strain increasing too? The cynic would then say that you don’t need the complication of DIC. So I think you can emphasise more strongly that, while the RSG gives the same indication of increasing damage through the strain magnitude increase, that ( superior to RSG) the DIC allows location detection also.  Just the addition of a couple of words here would emphasise the advantages of DIC for this sort of work.

**We have rephrased the text as follows: Based on the correlation between data from DIC and RSG, we found that the prototype DIC system exhibited an excellent performance for damage detection and stability in tests over long times. In addition to the onset of damage propagation, the full-field deformation maps from DIC provide information about the location of the damage in a defined RoI.**

 Also please be consistent with terminology – suggest you refer only to “increasing compressive strain” and don’t then talk about decreasing strain.

**We have implemented this change to the text.**

 Final line of Results and discussion section: never start a sentence with “And”.

**We have rephrased the sentence.**
